# Biallelic variants in *SUPV3L1* cause a variable leukodystrophy due to impaired mitochondrial degradosome function

**DOI:** 10.21203/rs.3.rs-4356120/v2

**Published:** 2026-07-08

**Authors:** Lydia Green, Noémie Hamilton, Marilena Elpidorou, Reza Maroofian, Maha S. Zaki, Andrew G.L. Douglas, Katrin Õunap, Ailsa M.S. Rose, Erica L. Harris, Diogo Candeias, Stone Elworthy, Stephen A. Renshaw, Elizabeth C. Low, David H. Dockrell, Kristian Tveten, Geoffrey Wells, Sarah A. Harris, Almundher Al-Maawali, Khalid Al-Thihli, Sana Al-Zuhaibi, Amna Al Futaisi, Daniel G. Calame, Ivan K. Chinn, Kristen S. Fisher, Mario Sa, Daniel Warren, Mina Zamani, Saeid Sadeghian, Reza Azizimalamiri, Hamid Galehdari, Gholamreza Shariati, Tahere Seifi, Erum Afzal, Mark A. Tarnopolsky, Lauren Brady, Stephan Zuchner, Niloofar Chamanrou, Annarita Scardamaglia, Emma Wakeling, Prab Prabhakar, Carla Roca-Bayerri, Gillian I. Rice, Clément Prouteau, Céline Bris, Marine Tessarech, Inger Sandvig, Noureddine Hedjem, Aaisha Al Balushi, Dana Marafi, Henry Houlden, Eamonn G. Sheridan, Colin A. Johnson, John H. Livingston, Yanick J. Crow, James A. Poulter

**Affiliations:** 1 Leeds Institute of Medical Research, University of Leeds, Leeds, UK; 2 Department of Paediatric Neurology, Leeds Teaching Hospitals Trust, Leeds, UK; 3 The Bateson Centre, Division of Clinical Medicine, University of Sheffield, Sheffield, UK; 4 York Biomedical Research Institute, Department of Biology, University of York, YO10 5DD, York, UK; 5 Department of Neuromuscular Disorders, University College London, London, UK; 6 Clinical Genetics Department, Human Genetics and Genome Research Institute, National Research Centre, Cairo, Egypt; 7 Oxford Centre for Genomic Medicine, Oxford University Hospitals NHS Foundation Trust, Oxford, UK; 8 Nuffield Department of Clinical Neurosciences, University of Oxford, Oxford, UK; 9 Department of Clinical Genetics and Personalised Medicine Clinic, Institute of Clinical Medicine, University of Tartu, Tartu, Estonia; 10 Genetics and Personalised Medicine Clinic, Tartu University Hospital, Tartu, Estonia; 11 Florey Institute, School of Medicine and Population Health, University of Sheffield, Sheffield, UK; 12 Institute for Regeneration and Repair, University of Edinburgh, Scotland, UK; 13 Department of Medical Genetics, Telemark Hospital Trust, Skien, Norway; 14 UCL School of Pharmacy, University College London, 29/39 Brunswick Square, London, UK; 15 Astbury Centre for Structural Molecular Biology, School of Physics and Astronomy, University of Leeds, Woodhouse Lane, Leeds, UK; 16 Department of Genetics, College of Medicine and Health Sciences, Sultan Qaboos University Hospital, University Medical City and Sultan Qaboos University, Oman; 17 Ophthalmology Department, College of Medicine and Health Sciences, Sultan Qaboos University Hospital, University Medical City and Sultan Qaboos University, Oman; 18 Pediatric Neurology Unit, Department of Child Health, College of Medicine and Health Sciences, Sultan Qaboos University Hospital, Sultan Qaboos University, Oman; 19 Section of Pediatric Neurology and Developmental Neurosciences, Department of Pediatrics, Baylor College of Medicine, Houston, TX, USA; 20 Division of Immunology, Allergy and Retrovirology, Department of Pediatrics, Baylor College of Medicine, Houston, TX, USA; 21 Paediatric Neurology Department, Oxford University Hospitals NHS Foundation Trust, Oxford, UK; 22 Department of Radiology, Leeds Teaching Hospitals Trust, Leeds, UK; 23 Narges Medical Genetics and Prenatal Diagnosis Laboratory, Kianpars, Ahvaz, Iran; 24 Department of Biology, Faculty of Science, Shahid Chamran University of Ahvaz, Ahvaz, Iran; 25 Department of Pediatric Neurology, Golestan Medical, Educational, and Research Center, Ahvaz Jundishapur University of Medical Sciences, Ahvaz, Iran; 26 Department of Medical Genetics, Faculty of Medicine, Ahvaz Jundishapur University of Medical Sciences, Ahvaz, Iran; 27 Developmental-Behavioral Pediatrics, Children Hospital and the Institute of Child Health Multan, Pakistan; 28 Department of Pediatrics, McMaster University, Hamilton, ON, Canada; 29 Department of Human Genetics, University of Miami Miller School of Medicine, Miami, FL, USA; 30 Department of Genetics, Faculty of Science, Shahrekord University, Shahrekord, Iran; 31 Department of Biology, Faculty of Science, Shahid Chamran University of Ahvaz, Ahvaz, Iran; 32 North East Thames Regional Genetic Service, Great Ormond Street Hospital for Children NHS Foundation Trust, London, UK; 33 Department of Paediatric Neurology, Great Ormond Street Hospital for Children NHS Foundation Trust, London, UK; 34 Centre for Genomic and Experimental Medicine, Institute of Genetics and Cancer, University of Edinburgh, Edinburgh, UK; 35 Division of Evolution and Genomic Sciences, School of Biological Sciences, University of Manchester, Manchester Academic Health Science Centre, Manchester, UK; 36 Department of Genetics, Angers University Hospital, Angers, France; 37 Department of Child Neurology, Oslo University Hospital, Oslo, Norway; 38 Neonatal Care, Perpignan Hospital, Perpignan, France; 39 National Genetic Center, Royal Hospital, Muscat, Oman.; 40 Department of Pediatrics, Faculty of Medicine, Kuwait University, Kuwait; 41 MRC Human Genetics Unit, Institute of Genetics and Cancer, University of Edinburgh, Edinburgh, UK; 42 Laboratory of Neurogenetics and Neuroinflammation, Institut Imagine, Paris Descartes University, Paris, France

## Abstract

**Purpose:**

Sensing and degradation of double stranded RNA (dsRNA) in the cell is tightly regulated to avoid activation of type I interferon signalling. The mitochondrial dsRNA degradosome complex is formed by PNPT1 and SUPV3L1. While biallelic *PNPT1* mutations are an established cause of early-onset encephalopathy, the clinical and radiological impact of SUPV3L1 dysfunction is yet to be fully defined.

**Methods:**

Through an international collaboration, we identified 21 patients with biallelic *SUPV3L1* mutations. Available clinical and radiological data were compared. A *supv3l1* knock-out zebrafish was generated to investigate the impact of *supv3l1* loss *in vivo*.

**Results:**

Fifteen different biallelic loss-of-function *SUPV3L1* variants were identified in twenty-one individuals presenting with a wide clinical spectrum including neonatal haematological disturbance, infant-onset motor disorder and acute encephalopathy. Most individuals demonstrated significant neurodevelopmental involvement, manifesting as moderate to severe motor delay with intellectual impairment. Other common clinical features include microcephaly and spasticity. Three out of four patients tested showed an increased interferon signature in peripheral blood. A *supv3l1* knock-out zebrafish exhibited defective mitochondria morphology and microglial function, with a significant activation of type 1 interferon signalling.

**Conclusion:**

We define the genetic, clinical and radiological spectrum of *SUPV3L1*-asociated disease and suggest activation of the type 1 interferon innate immune pathway by dysplastic microglia as a possible underlying cause.

## Introduction

Inherited white matter disorders (IWMDs) result from disruption of any component of white matter structure or function.^1,2^ Advances in magnetic resonance imaging (MRI) and genomic medicine have led to the identification of multiple leukodystrophy (LD) and LD-associated genes. As well as classical primary IWMDs, where disruption of the myelin network is the primary pathology, an increasing number of secondary LDs have been described where the impact is consequent upon another, potentially systemic, disease process. There are now growing numbers of heterogenous neurodevelopmental phenotypes associated with evidence of abnormal white matter. Approximately 10% of these are in mitochondria-associated genes.^3^ While many impact Complexes I-IV, a growing proportion of these disorders result from disturbance of cellular metabolism, proliferation and innate immune signalling.^4–6^

The mitochondrial degradosome is a functionally conserved complex composed of the ATP-dependent RNA and DNA helicase SUV3 or SUPV3L1 (encoded by *SUPV3L1*) and the PNPase PNPT1 (encoded by *PNPT1*). The degradosome plays a key role in maintaining mitochondrial integrity through surveillance and degradation of double stranded (ds) RNA. In addition to an accumulation of R loops in the mitochondrial genome, with consequent disruption of replication and mitochondrial genome integrity, loss of degradosome activity due to *PNPT1* mutations has been shown to result in leakage of mitochondrial dsRNA into the cytosol and activation of the antiviral immune response.^7,8^ While biallelic *PNPT1* variants have been extensively reported,^9–11^ much less is known about the clinical and cellular phenotypes associated with pathogenic variants in *SUPV3L1*.

Three studies recently reported a total of five patients with biallelic *SUPV3L1* variants; a sibling pair with optic atrophy, skin hypopigmentation and progressive neurodegeneration, a second sibling pair with global developmental delay, seizures and white matter abnormalities, and a singleton manifesting episodic neuroregression, spasticity, optic atrophy and skin hypopigmentation. One set of siblings were homozygous for a *SUPV3L1* stop mutation (c.2215C>T; p.(Gln739Ter))^12^, the second sibling-pair homozygous for *SUPV3L1* (c.1093C>T; p.(Arg365Trp))^13^, and the singleton compound heterozygous for a c.272–2A>G variant in combination with an amino acid substitution (c.1924A>C; p.(Ser642Arg)).^14^

Here we describe twenty individuals from fourteen families with biallelic *SUPV3L1* variants and define the clinical and radiological spectrum. Using a knockout zebrafish model, we demonstrate that loss of SUV3 function results in altered mitochondrial biogenesis, dysplastic microglia and activation of type 1 interferon signalling. Thus, we define the helicase SUV3 as a critical player in neurodevelopment.

## Methods

### Patient ascertainment

We ascertained twenty patients from fourteen unrelated families across thirteen institutions using GeneMatcher.^15^ Biallelic *SUPV3L1* variants were identified locally by next generation sequencing techniques following local procedures. Clinical and radiological data were collected retrospectively in Leeds and evaluated by LG and DW. The study was approved by the Yorkshire & Humber – Leeds East Research Ethics Committee (REC ref. 18/YH/0070). All patients and/or parents gave appropriate informed consent to be part of this study, and to be included in any publication, from their local institution.

### Molecular and cellular assays

#### Plasmid preparation and validation

Gene blocks of *att*B flanked *SUPV3L1* containing each missense variant tested were obtained from Integrated DNA Technologies (Coralville, Iowa). Gene blocks were cloned into pDONR221 donor vector using the Gateway^™^ BP Clonase^™^ II Enzyme mix (ThermoFisher Scientific) and further cloned into the required destination vector using the Gateway^™^ LR Clonase^™^ II Enzyme mix (ThermoFisher Scientific) according to the manufacturer’s protocol. Sanger sequencing of the vectors confirmed the presence of each variant.

#### SUPV3L1 complementation assay

2.5×10^5^ U2OS cells were seeded on to coverslips and 24 hours later transfected with a custom siRNA targeting the 5’UTR of *SUPV3L1* (Horizon technologies) using lipofectamine RNAiMAX (Thermo Fisher Scientific). 24 hours later, a second transfection was performed to introduce wild-type or variant-containing *SUPV3L1* using TransIT-X2 (Mirrus Bio). After 48 hours, U2OS cells were fixed with 4% paraformaldehyde. Following fixation, cells were permeabilised with 0.02% Triton X-100 for 5 minutes before washing with 1x phosphate-buffered saline (PBS) for a further 5 minutes. Wells were then blocked with 5% milk:PBS solution for 60 minutes at room temperature. Primary anti-dsDNA antibody (K1; Cell Signalling Technology) was prepared at a 1:500 dilution in a 3% milk:PBS solution and added to the wells for 60 minutes at room temperature. After three washes with PBS, cells were incubated with an Alexa Fluor 488 anti-mouse IgG secondary antibody (Thermo Fisher Scientific, 1:500) and Hoescht (1:1,000). Secondary antibodies were prepared with 1% milk in PBS and incubated at room temperature, out of light, for 60 minutes. Once completed the cells were washed three times with PBS before mounting. Images were captured using a Nikon A1R confocal microscope using NIS Elements software. ImageJ software was used to count dsRNA, using the SpotCounter plugin.

### Zebrafish Modelling

#### Zebrafish husbandry and ethics

All zebrafish were raised in the Bateson Centre at the University of Sheffield in UK Home Office approved aquaria and maintained following standard protocols^16^. Tanks were maintained at 28°C with a continuous re-circulating water supply and a daily light/dark cycle of 14/10 hours. All procedures were performed under an animal Project Licence to standards set by the UK Home Office. We used the Tg*(mpeg1:mCherryCAAX)*sh37*8* labelling the membrane of macrophages and microglia^17^ and the Tg(*AnnexinV*:mVenus)sh632^18^ to label dying cells.

#### Generation of supv3l1 zebrafish crispants

Synthetic SygRNA^®^ consisting of gene-specific CRISPR RNAs (crRNA) (Sigma) and transactivating RNAs (tracrRNA) (Merck) in combination with CAS9 nuclease protein (Merck) was used for gene editing. TracrRNA and crRNA were resuspended to a concentration of 100μM in nuclease free water containing 10mM Tris-HCl ph8. SygRNA^®^ complexes were assembled on ice immediately before injection using a 1:1:1 ratio of crRNA:tracrRNA:Cas9 protein. We used the CHOPCHOP website^19^ to design the following crRNA sequence specific to the zebrafish *supv3l1* gene (ENSDARG00000077728) targeting exon1, where the PAM site is indicated in brackets: GAAGACGCGGAGGGATCAGT(CGG). A 0.5nl drop of SygRNA^®^:Cas9 protein complex was injected into one-cell stage embryos. The resulting *supv3l1* crispants were used for the experiments, alongside a scrambled crRNA sequence for control group^20^.

#### Construction of Tg(mpeg1.1:mts-mNeonGreen)sh631

The Tol2kit multisite Gateway method was used with p5E-mpeg1.1^21^, pME-mts-mNeonGreen, p3E-polyA and pDestTol2pA2^22^ to create p(mpeg1.1:mts-mNeonGreen) transgene plasmid. The pME-mts-mNeonGreen middle-entry plasmid incorporated mts-mNeonGreen PCR amplified from a custom gBlock from IDT. The mts-mNeonGreen had the mitochondrial targeting sequence from zmLOC100282174^23^ as an N-terminal fusion to mNeonGreen^24^ via a (GGGGS)3 flexible linker and was codon optimised using CodonZ3^25^. DNA sequencing confirmed the full transgene plasmid sequence (Core Genomics Facility University of Sheffield). The transgene plasmid was co-injected with tol2 transposase mRNA into Zebrafish embryos, the fish raised to adulthood and 3-day post fertilisation (dpf) larvae from outcrosses were screened for green fluorescent macrophages using a Zeiss Axio ZoomV16 microscope. Such F1 larvae were raised to adulthood and screened by outcrossing. This identified the line sh631 that transmitted the transgene to 50% of progeny, indicating a single transgenic insertion.

#### Quantitative PCR

RNA was extracted from dissected heads of 5-day post fertilisation (dpf) zebrafish larvae using the Trizol/chloroform method. cDNA was synthetised using the SuperScript II kit (Invitrogen) with 2mg of RNA following manufacturer instructions and diluted in 1:20 for qPCR. qPCR primers were tested for efficiency (85%−105%) using a cDNA serial dilution. The qPCR reaction was run in a CFX96 Bio-Rad machine using validated primers as previously described^20^. Expression was normalised to *rpl13* as reference gene and relative to scrambled control set to 1.

#### Zebrafish confocal imaging and analysis

To assess microglial morphology, 5dpf scrambled and *supv3l1* crispant larvae injected in double reporter background Tg*(mpeg1:mCherryCAAX)*sh378 and Tg(*AnnexinV:mVenus*)sh632 were anaesthetised and embedded in low melting point agarose containing tricaine (0.168 mg/ml; Sigma-Aldrich) and imaged using a 40x objective on a UltraVIEW VoX spinning disk confocal microscope (PerkinElmer Life and Analytical Sciences). Z-stacks were 100μm thick using 0.5μm slices. For microglial morphology analysis, sub-stacks of 50μm were used to create a maximum projection and contours of each microglia (avoiding pigment cells) were drawn using a pen tablet (Intuos from Wacom). Using Fiji, the circularity index (0–1) of each microglia was automatically recorded to assess the circularity of each cell with the value of 0 being not circular and 1 as being perfectly circular.

To count the number of apoptotic cells, the same animals were imaged using the 10x lens on a UltraVIEW VoX spinning disk confocal microscope (PerkinElmer Life and Analytical Sciences) using the brightfield alongside GFP and DsRed channels. Z- Stacks of 100μm with 2μm per slice were acquired and the brightfield image was used to draw a region of interest around the optic tectum as previously described^20^.

#### TUNEL staining and quantification

Fish larvae at 5dpf were fixed in 4% PFA and the TUNEL assay was performed according to standard protocol using the ApopTag Kit (Millipore) and as previously described^20^. Samples were imaged on the inverted W1 Nikon Spinning Disk confocal microscope using the brightfield and GFP channel by acquiring stacks of approximately 100μm with 2μm per slice. Using Fiji, the optic tectum region was outlined with the free hand drawing tool using the brightfield image and counting of number of apoptotic cells was performed using the automatic thresholding and particle count. All imaging analysis were blinded until after counting was performed.

### Statistical analysis

All statistical analyses were performed in GraphPad Prism where data was entered using a column (2 samples, 1 variable only) spreadsheet. Sample distribution was assessed using frequency of distribution analysis and two-tailed Mann-Whitney *U* test was used for statistical analysis. All experiments were repeated using different batches of larvae born on different dates, with the number of biological replicates and n (experimental unit) number stated for each experiment in figure legends. p values are indicated and a star system is used instead for graph with multiple comparisons: *=p<0.05, **=p<0.01, ***=p<0.001, ****=p<0.0001. Following the recommendation of the American Statistical Association we do not associate a specific p value with significance^26^.

## Results

We identified fifteen different *SUPV3L1* variants segregating with disease in 21 individuals. All variants are rare (minor allele frequency < 0.001 in gnomAD v4.0) and were considered likely pathogenic by in silico analysis (CADD v1.7 score > 15, predicted damaging (PolyPhen2), deleterious (SIFT) or to impact splicing (SpliceAI > 0.4)) (Table 1). Variants identified included missense, frameshift, nonsense and splice site alterations. All amino acid residues in which a missense variant was identified showed a high degree of evolutionary conservation (Supplementary Figure 1).

### Biallelic *SUPV3L1* mutation causes a variable neurodevelopmental phenotype

To better understand the clinical impact of biallelic *SUPV3L1* mutations, we collated the phenotypic data relating to all twenty-one identified patients (detailed clinical characteristics provided in Supplementary Table 1). All patients presented in childhood, with onset under 12 months in all cases where data was available, the majority with developmental delay (15/21). Three patients presented prior to any noted delay, one in the antenatal period with abnormal antenatal scans, one at birth with neonatal thrombocytopenia and hypoglycaemia, and one postnatally with feeding issues and weight loss. No other patients had haematological or biochemical abnormalities reported. Other features at presentation included hypotonia (7/21), nystagmus (4/21), acquired microcephaly (1/21) and ambiguous genitalia (1/21). P12 presented aged 4 years with acute encephalopathy, ataxia and dystonia following a viral illness but, prior to this, development was normal except for mild speech articulation difficulties. At the time of writing one patient (P11) was deceased - antenatal scans demonstrated lissencephaly in this individual, who experienced respiratory distress syndrome, neonatal seizures and early spasticity.

Over time all surviving patients demonstrated some degree of developmental delay, being described as severe in 5/21, and with 15/21 manifesting clear intellectual disability. At last review 19/21 had spasticity, 16/21 microcephaly, 9/21 areas of skin hypopigmentation, 6/21 seizures, 2/21 movement disorders and 2/21 retinal dystrophy or unspecified retinal changes. Twelve patients achieved walking, although only five independently. The seven others continue to require significant support aged between 4–20 years.

### *SUPV3L1*-associated disorder has a variable radiological phenotype including abnormal white matter, temporal pole cysts and calcification

**Figure 1:**
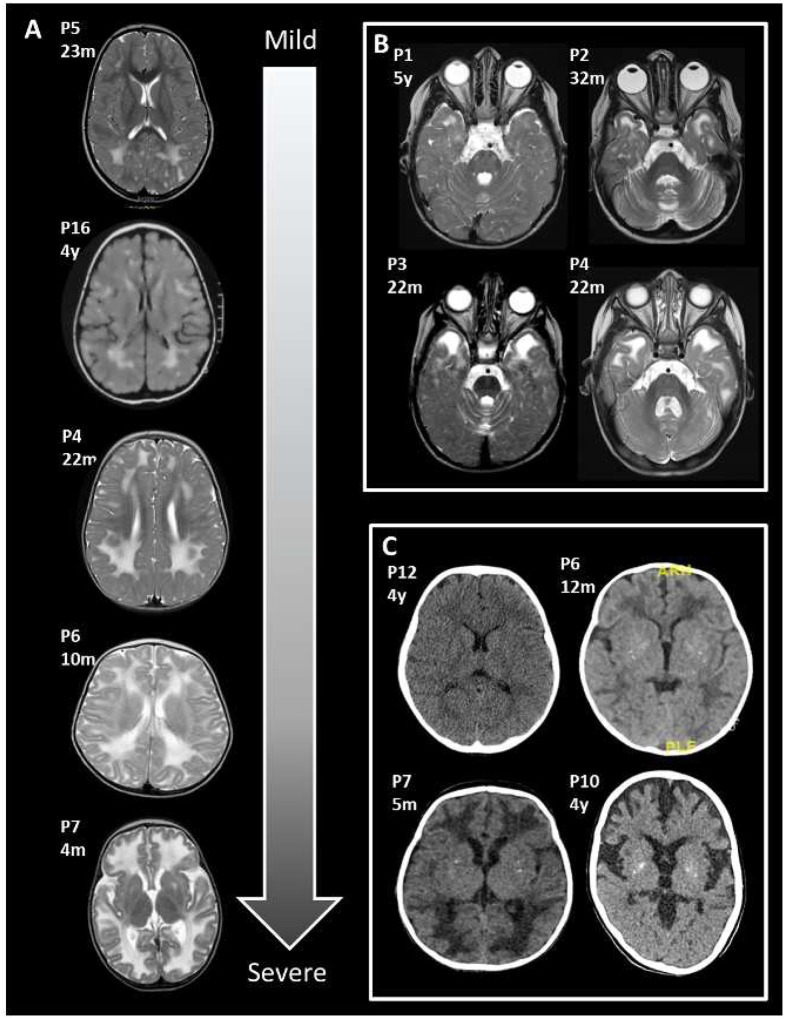
Spectrum of MRI findings in selected patients with *SUPV3L1*. Axial T2 imaging identified variable degrees of white matter abnormality in 12/15 patients (A) and temporal pole cysts in 5/15 (B). Other common features included cerebral and cerebellar atrophy. CTH was available in 4 patients (C), 3 of whom demonstrated small foci of calcification in the basal ganglia.

**Figure 2:**
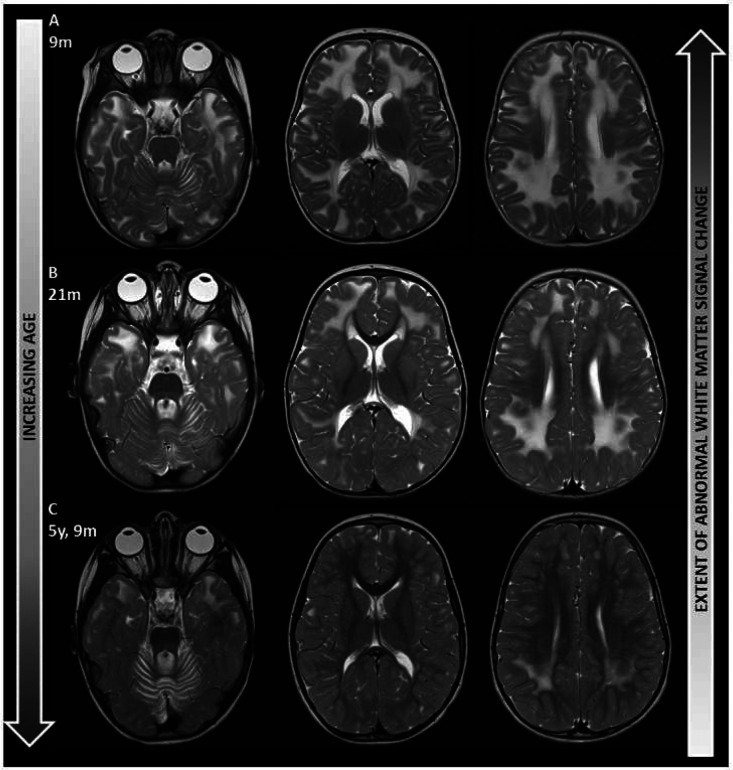
Follow up imaging demonstrating progressive cerebellar atrophy and improvement in abnormal T2 signal. Axial T2 imaging for P4 performed at 9 months (A), 21 months (B) and 5 years, 9 months (C) demonstrating temporal horn cysts, progressive cerebellar atrophy and unexpected improvement in leukodystrophy. Over a 5-year period she progresses from extensive bilateral, confluent T2 signal hyperintensity in the deep and subcortical white matter, to more subtle T2 hyperintensity with posterior predominance and less prominent cystic change at the temporal poles. There was no waxing or waning in clinical presentation.

Imaging was available for 15 of the 21 patients (14 MRI, 1 foetal MRI, 4 CT head). MRI and CT reports alone were available for P20 and P21 respectively. Detailed MRI findings are available in Figures 1, 2 and S2. White matter was abnormal in 11/16 patients, ranging from mild delay through to confluent, bilateral T2 hyperintensity. In the only patient with follow up imaging available, there was an improvement in T2 signal over time. Subcortical cysts in the temporal pole were seen in five patients. This feature could not be assessed in P5–7 due to incomplete imaging. Additional features included atrophy (cerebellar 11/16, cerebral 5/16) and abnormality of the corpus callosum (5/16). Of the four patients with available CT imaging, three had small foci of calcification within the basal ganglia.

P11 presented antenatally, with an MRI at 28 weeks of gestation demonstrating lissencephaly, partial agenesis of the corpus callosum, agenesis of the cerebellar vermis, pons hypoplasia, posterior fossa cyst and multiple subcortical white matter cystic lesions. A further scan at age 3 days (not available for review) was said to have confirmed the antenatal findings with no additional features recorded. P12 had normal MRI imaging, both at presentation and following a second episode of regression at age 6 years.

### Impact of *SUPV3L1* variants on SUV3 function

Mapping of the identified variants onto SUV3 in 2D and 3D (Figure 2A, B) revealed only 3/15 mapped to a known functional domain (p.Alal393Thr, p.Pro414Ser and p.Tyr441LeufsTer10), with these three all located in the helicase domain. The impact of the 12 remaining variants, located outside of any functional domains, is predicted to be structural. To assess pathogenicity, a cell-based functional assay was performed using the amount of intracellular dsRNA as a functional readout (Supplementary Figure 3), which confirmed all variants significantly impacted SUV3 protein function and are likely pathogenic (Figure 2C).

### *SUPV3L1* mutations result in variable induction of type I interferon signalling

mtdsRNA are known immunogenic substrates and have been shown to activate the antiviral immune response when accumulated in patients with pathogenic *PNPT1* variants. A previous study, however, did not observe a response following SUV3 depletion, presumed due to restriction of mtdsRNA to the mitochondria.^8^ Assessment of interferon signalling status in the blood of four patients (P4, P6, P7, P12) revealed a marked up-regulation of interferon stimulated genes (ISGs) in three patients (Supplementary Figure 4). P6 was tested on two occasions, showing a positive response initially, though less marked than her sibling, followed by a subsequent negative response. The role of aberrant SUV3 in innate immune activation, at least as reflected by ISG expression in blood, is therefore variable and may change over time.

### Loss of *supv3l1* in a zebrafish model results in immune dysfunction and activation of antiviral innate immune signalling

**Figure 4:**
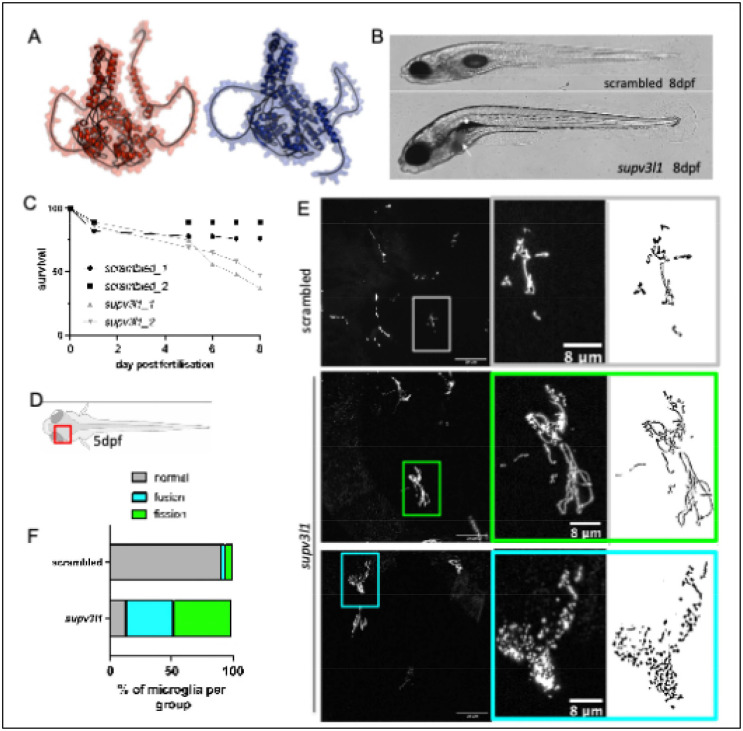
Loss of *supv3l1* in zebrafish decreases survival and induces mitochondrial stress. A. Ray traced diagrams of the human SUPV3l1 (red, UniProt: Q8IYB8) and zebrafish Supv3l1 (blue, UniProt: A4IG62) predicted structures, 3D structural alignment was generated with PyMol using the ‘align to molecule’ function (RMSD = 0.313 Å). B. Representative brightfield images of 8dpf zebrafish larvae injected with scrambled control and *supv3l1* crRNA. Note the necrotic liver (white arrow) and deflated swim bladder (white asterisk) in *supv3l1* injected larvae as previously reported in *supv3l1* mutant. C. Survival analysis of scrambled control and *supv3l1* crRNA injected larvae until 8dpf. Death outcome is represented by humanely culling animals displaying an emaciated phenotype. D. Diagram of a dorsal view of a 5dpf zebrafish head with red box highlighting the region imaged and the transgenic lines used in panel E. E. Representative confocal images of 5dpf zebrafish brains from scrambled control and *supv3l1* crRNA injected larvae in the macrophage-specific mitochondria reporter line Tg(mpeg1:mls-neon)^*SH631*^. Scale bar 29μm. High magnification image of a microglia selected from the coloured rectangle and associated Mask image generated in FiJi using the ‘Tubeness’ plugins. Scale bar 8μm. F. Quantification of number of microglia displaying a normal, fusion or fission mitochondria phenotype in the optic tectum. n=12–14 from 2 independent experiments, using Chi-square test from contingency table (****: p<0.0001).

At a protein structural level, zebrafish supv3l1 is highly homologous to human SUV3 (Figure 4A). To investigate the effect of *SUPV3L1* loss of function on brain development *in vivo*, we generated a *supv3l1* crispant using a CRISPR/Cas9 approach and assessed the impact on survival and mitochondrial function, which have both been shown to be affected by *supv3l1* loss-of-function in zebrafish^27^. Embryos injected with *supv3l1* crRNA showed reduced survival compared to scrambled-injected controls, necessitating culling by 8dpf due to signs of liver necrosis, lack of swimming and deflated swim bladder (Figure 4B,C). Mitochondrial dysfunction was confirmed using a novel reporter line Tg(*mpeg1*:mls-neon)sh631 labelling mitochondria in the macrophage/microglia lineage, with significantly more microglia displaying mitochondria fusion and fission in *supv3l1* crispants (Figure 4D–F).

**Figure 5:**
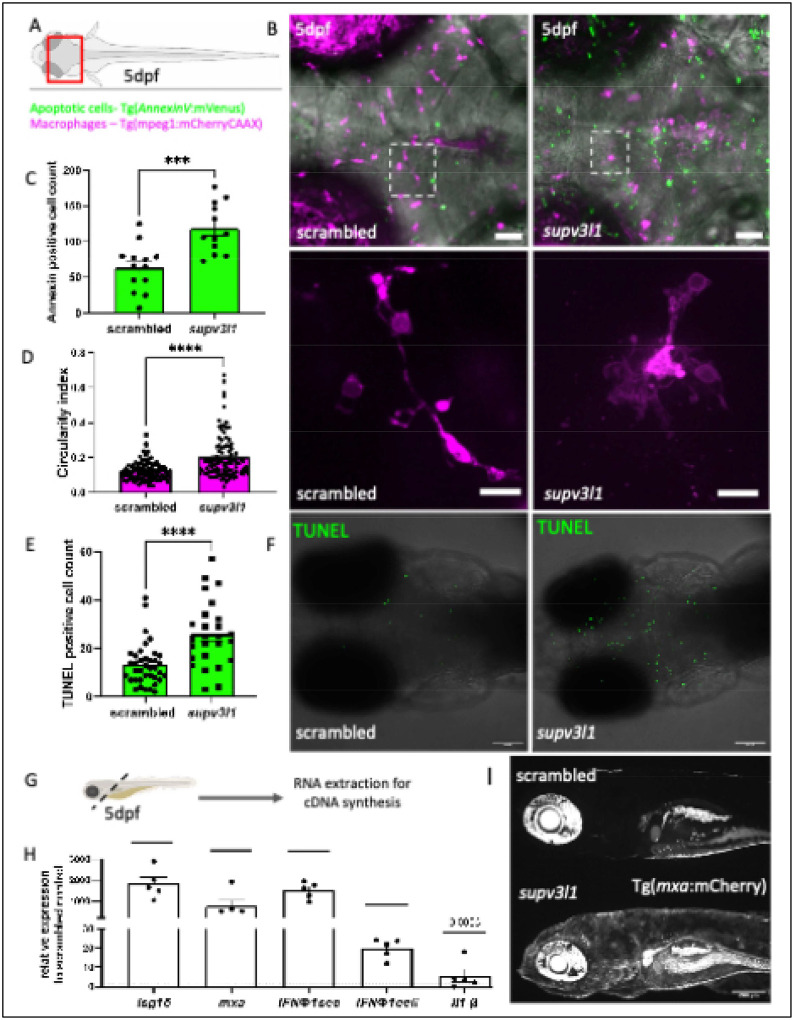
Loss of *supv3l1* activates microglia and triggers systemic antiviral immunity activation in zebrafish. **A.** Diagram of a dorsal view of a 5dpf zebrafish head with red box highlighting the region imaged and the transgenic lines used in panel F. **B.** Representative confocal images of 5dpf zebrafish brains from scrambled control and *supv3l1* crRNA injected larvae in the double Tg(*AnnexinV*:mVenus)sh632;Tg(*mpeg1*:mCherryCAAX)sh378 background, labeling apoptotic cells and macrophages respectively. Scale bar 50μm. High magnification image of a macrophage selected from the dotted rectangle. Scale bar 10μm. **C.** Quantification of Annexin positive apoptotic cells in the optic tectum. n=12–13 from 2 independent experiments, using two-tailed Mann-Whitney U test (***: p<0.001). **D.** Quantification of macrophages circularity measuring the circularity index using the Tg(*mpeg1*:mCherryCAAX)sh378 reporter line. n=98–115 macrophages from 3 independent experiments, using two-tailed Mann-Whitney U test (****: p<0.0001). **E.** Quantification of TUNEL positive apoptotic cells in the optic tectum. n=26–38 from 3 independent experiments, using two-tailed Mann-Whitney U test (****: p<0.0001). **F.** Representative confocal images of 5dpf zebrafish brains from scrambled control and *supv3l1* crRNA injected larvae and stained for apoptotic cells using TUNEL staining. Scale bar 100μm. **G.** Diagram of experimental setup to analysis gene expression from zebrafish brain by qPCR **H.** Representative confocal images of 5dpf zebrafish from scrambled control and *supv3l1* crRNA injected larvae in the Tg(cryaa:Dsred;mxa:mCherryF)^*ump7tg*^ reporter line. Scale bar 200μm. **I**. qPCR analysis of antiviral genes *mxa*, *isg15*, ifnΦ1 secreted and cellular, and inflammatory gene *il1-beta* from heads of 5dpf scrambled control and *supv3l1* crRNA injected larvae. Expression relative to scrambled control set at 1 (dotted line) and normalised to *rpl13* reference gene. n=5 from 5 independent experiments, two-tailed Mann-Whitney U test, p value shown on individual graphs.

To analyse the effect of dysfunctional mitochondria in microglia and the wider brain, 5dpf zebrafish were live imaged at high resolution to measure microglial morphology and cell death in the optic tectum region, using Tg(*mpeg1*:mCherryCAAX)sh378 and Tg(*AnnexinV*:mVenus)sh632 reporter lines respectively. Microglia in brains of 5dpf *supv3l1* crispants displayed an activated phenotype, with a rounder cell body (Figure 5A–D), and showed an unusual membrane ruffled phenotype (Figure 5B- high magnification panel). Using the Tg(*AnnexinV*:mVenus)sh632 reporter line to label apoptotic cells, we counted a higher number of apoptotic cells in *supv3l1* crispant brains, which was confirmed using TUNEL staining (Figure 5E,F).

To assess the neuroinflammatory state of the brain, qPCR of a panel of inflammatory genes was performed on RNA from heads of 5dpf *supv3l1* and control fish (Figure 5G,H). We observed a >400-fold upregulation of expression of genes specific to the antiviral immune pathway, including the human orthologue of IFN-1 called *ifnF1*^28^ and interferon-induced genes such as *isg15* and *mxa* (Figure 5H). Activation of the antiviral immune response was confirmed using a reporter line for the *mxa* gene Tg(*cryaa*:Dsred;*mxa*:mCherryF)ump7tg, highlighting a systemic activation of antiviral immune pathways (Figure 5I).

## Discussion

DNA/RNA helicases are highly conserved motor proteins with a role in almost all processes involving nucleic acid replication, repair and degradation.^29^ Maintaining effective turnover of unwanted nucleic acid material is critical for mitochondrial health and function. SUV3, encoded by *SUPV3L1*, is an ATP-dependent RNA/DNA helicase that, when bound to polyribonucleotide nucleotidyltransferase 1 (PNPT1 or PNPase), forms an essential part of the human mitochondrial degradosome.^30–32^ If one of the two components of the heteropentamer is disrupted, the activity of the degradosome is abolished. While biallelic variants in *PNPT1* have been associated with abnormal myelination in patients, neurodevelopmental delay and mitochondrial dysfunction,^8,33^ our study provides additional evidence that pathogenic variants in *SUPV3L1* can result in neurodevelopmental disturbances, characterised by defects in mtdsRNA processing and function.

The first cases of *SUPV3L1* mutations causing human disease were reported by Dawidziuk *et al*. in a pair of siblings with microcephaly, one of whom had white matter changes.^13^ Van Esveld *et al*. reported a further two cases where *SUPV3L1* variants were considered responsible for progressive neurodegeneration in a sibling pair, with a family history of two other affected siblings and an affected cousin from whom samples were not available for testing.^12^ The siblings were homozygous for the same c.2215C>T, p.Gln739* variant, which was shown by Esveld *et al*. to produce a truncated SUPV3L1 protein, identified in three families in our cohort (F6, F7 and F14), all of whom were ascertained from the Middle East.^34^ Tsygankova *et al*. more recently reported a 17 year old with episodic regression, leading to spastic paraparesis, intellectual disability and optic atrophy. While these initial reports implicated pathogenic *SUPV3L1* variants as a cause of disease, neither the clinical/radiological phenotype nor the mechanism underpinning this disorder were well defined.

Through an international collaboration, our study reveals a wide clinical spectrum associated with *SUPV3L1*-related disease, from neonatal haematological disturbance through to infant-onset motor disorder and acute encephalopathy, with no apparent genotype-phenotype correlations. 18/21 individuals in our cohort demonstrated a significant neurodevelopmental disorder, manifesting as moderate to severe motor delay with intellectual impairment. Other common clinical features include microcephaly and spasticity. Nine patients (42%) manifest areas of skin hypopigmentation, at least two being consistent with vitiligo and showing progression over time. Hypopigmentation occurs secondary to defects in melanin production, with causes including post-infectious/inflammatory, post-traumatic and autoimmune responses. A third of the cohort experienced one or more episodes of developmental regression, often without subsequent full return of function. Episodes of regression, often on a background of preceding developmental delay, are a recognised feature of disorders of mitochondrial function.^35^

Imaging features of *SUPV3L1*-related disorders include abnormal white matter, ranging from mild, patchy T2 hyperintensity through to confluent bilateral abnormalities, anterior temporal horn cysts and basal ganglia calcification. Anterior temporal subcortical cysts are reported in both congenital infections (cytomegalovirus and rubella) but also other leukodystrophies and *RMND1*-related mitochondrial leukoencephalopathy.^36,37^ Intracranial calcification is seen in a number of other leukodystrophies, most notably Aicardi-Goutières syndrome (AGS). This observation, along with neonatal thrombocytopenia, meant that AGS was the initial differential diagnosis in P6, but extended genetic screening of AGS-associated genes was negative.

Three patients (P11, P12 and P18) represent outliers in our cohort, in terms of clinical and radiological presentation. P11 presented antenatally with significant abnormalities on MRI imaging at 28 weeks of gestation and died age 25 days due to respiratory distress and neonatal seizures. In addition to the homozygous p.Ala393Thr in SUV3, exome sequencing identified a heterozygous *TUBB3* (NM_006086.4:c.1243A>G, p.(Met415Val)) variant inherited from a healthy father and therefore presumed non-pathogenic. Poirier *et al*. reported twelve patients with mutations in *TUBB3* demonstrating cortical malformation, including polymicrogyria, gyral disorganization and simplification with or without pontocerebellar defects.^38^ Importantly, Poirier reported a mother and daughter in whom the heterozygous mother demonstrated only mild cognitive impairment, but had frontoparietal predominant gyral disorganisation and cerebellar vermis dysplasia indicating that even monoallelic *TUBB3* mutations can impact neuronal development. The *TUBB3* variant may therefore be contributing to the clinic-radiological phenotype in P11, accounting for the increased severity of their presentation.

Both P12 and P18 presented with regression following intercurrent illness aged 4 and 22 years respectively, on a background of mild developmental impairment. In neither case were any other candidate variants identified on trio exome sequencing. The *SUPV3L1* variants were predicted probably damaging and significantly impacted SUV3 function in our complementation assay. Although a milder phenotype, both presentations would be in keeping with an underlying mitochondrial disorder.

While all variants identified lead to loss of SUV3 function, complete loss of SUV3 is likely to be embryonic lethal. *Supv3l1* knockout in mice, results in embryonic lethality, with further investigation identifying a role for SUV3 in the cytoplasm to suppress mitotic homologous recombination.^30^ A gene trap *supv3l1* zebrafish mutant displayed mitochondrial dysfunction, impaired liver development and reduced survival, confirming a conserved role for SUV3 in vertebrate mitochondrial homeostasis.^27^ Altogether, this indicates *SUPV3L1* to be an essential gene with a fundamental role in degrading mtdsRNA. Our approach using *supv3l1* crispant zebrafish was consistent with the gene trap model, resulting in reduced survival.

dsRNA is an established pathogen-associated molecular pattern that can trigger the assembly of MDA5 ATP-sensitive filaments and activate the mitochondrial antiviral-signalling protein via CARD domain-mediated interactions.^39^ Genetic variants that cause impaired nucleotide metabolism have been reported and are well established aetiologies of other IWMDs such as due to mutations in *ADAR1*, *TREX1*, *SAMHD1*, *IFIH1* (which encodes MDA5), and *RNASEH2B*.^40–44^. The high incidence of skin hypopigmentation, also seen in three of the previously reported cases,^12^ referred to as vitiligo, could have an autoimmune origin linked to an enhanced antiviral immune response. mtdsRNA-mediated activation of the antiviral immune response therefore emerges as a possible pathophysiological mechanism in *SUPV3L1*-associated disease. Consistent with this hypothesis, and with pathogenic variants in *PNPT1*, activation of an antiviral immune response was observed *in vivo* in our zebrafish *supv3l1* model, and in keeping with the observation of increased ISG expression in blood of three of four patients in our cohort.

In summary, we describe the clinical and radiological phenotype of twenty one patients with a *SUPV3L1*-associated neurodevelopmental disorder. Modelling of *supv3l1* loss in fish demonstrated significant effects on mitochondrial biogenesis and microglial function with systemic activation of antiviral immune signalling. The role of SUV3 in the antiviral immune response opens the possibility of using immunomodulators as therapeutics to dampen the impact of pathogenic variants, particularly in cases with early normal development where repeated episodes of regression may lead to progressive neurological deterioration.

## Supplementary Material

Supplementary Tables

**Supplementary Table 1**: Clinical characteristics of eighteen affected individuals with biallelic *SUPV3L1* variants.

**Supplementary Table 2**: MRI features of patients with *SUPV3L1* variants.

Supplementary Figures

**Supplementary Figure 1**: Conservation of SUV3 missense variants in orthologs.

**Supplementary Figure 2:** Collated MRI imaging of patients with *SUPV3L1* variants.

**Supplementary Figure 3:** Representative images from the optimisation and validation of the dsRNA complementation assay.

**Supplementary Figure 4:** Interferon signature in patients with biallelic *SUPV3L1* mutations

Supplementary Files

This is a list of supplementary files associated with this preprint. Click to download.

- SUPV3L1manuscriptSUPPLEMENTARYMATERIALS.pdf

## Figures and Tables

**Figure 3:**
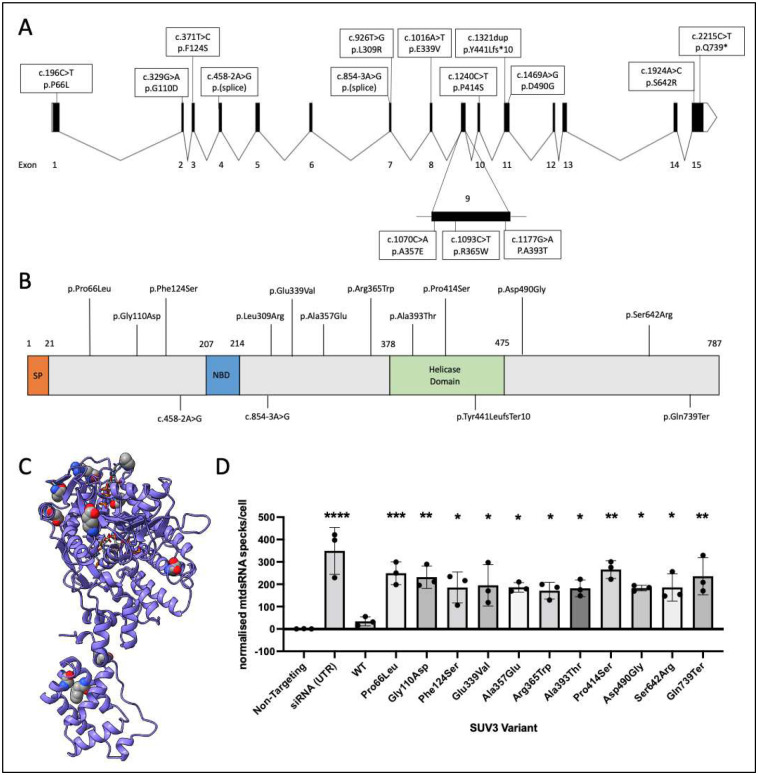
Genetic and proteomic mapping of pathogenic SUV3 variants. **A.** Schematic of the 15 exons and introns of human *SUPV3L1* annotated with the 15 different variants identified in this study**. B.** Schematic of the SUV3 protein showing the location of variants relative to the known protein domains. SP-Signal Peptide, NBD-Nucleotide Binding Domain **C**. Mapping of all missense variants on to the 3D crystal structure of SUV3 (protein backbone shown as a purple ribbon, mutation sties shown as spheres) **D.** Summary of complementation assay performed on all missense variants alongside a wild-type and disease (p.Gln793*) control. All missense variants identified failed to reduce the number of mtdsRNA to the levels of the wildtype, confirming an effect on SUV3 function.

**Table 1 –:** Summary of variants identified in *SUPV3L1*.

DNA variant	Protein variant	dbSNP: rs number	gnomAD v4.0 Frequency	PolyPhen2	SIFT	CADD (v1.7)	SpliceAI	Family
c.196C>T	p.Pro66Leu	rs752705209	1.8×10^−5^ (14/781,038)	Prob. Dam. 1.00	DEL 0.00	27.3	0.00	9*
c.329G>A	p.Gly110Asp	Not Present	Not Present	Benign 0.082	DEL 0.00	22.5	0.00	5
c.371T>C	p.Phe124Ser	Not Present	Not Present	Poss. Dam. 0.566	DEL 0.02	27.1	0.00	11
c.458-2A>G	p.(splice)	Not Present	1.4×10^−5^ (21/1,440,516)	N/A	N/A	32.0	0.99 (Acc.L)	2*
c.854-3A>G	p.(splice)	Not Present	Not Present	N/A	N/A	13.03	0.50 (Acc.L)	4
c.926T>G	p.Leu309Arg	Not Present	Not Present	Prob. Dam 0.996	DEL 0.00	32.0	0.00	13*
c.1016A>T	p.Glu339Val	Not Present	Not Present	Benign 0.085	DEL 0.01	26.8	0.46 (Don.L)	10
c.1070C>A	p.Ala357Glu	Not Present	Not Present	Prob. Dam. 0.959	DEL 0.00	26.4	0.00	2*
c.1093C>T	p.Arg365Trp	rs527626577	8.7×10^−6^ (14/1,613,968)	Poss. Dam. 0.457	DEL 0.00	33.0	0.00	1
c.1177G>A	p.Ala393Thr	rs1025320000	3.7×10^−6^ (6/1,613,896)	Prob. Dam. 0.936	DEL 0.00	29.5	0.01 (Don.G)	8
c.1240C>T	p.Pro414Ser	Not Present	1.6×10^−6^ (1/626,536)	Prob. Dam 0.968	DEL 0.00	26.2	0.00	9*
c.1321dup	p.Tyr441Leufs*10	Not Present	Not Present	N/A	N/A		0.00	3*
c.1469A>G	p.Asp490Gly	Not Present	Not Present	Prob. Dam. 0.995	DEL. 0.00	25.1	0.13 (Don.L)	10,12
c.1924A>C	p.Ser642Arg	rs750670256	5.0×10^−6^ (8/1,612,906)	Prob. Dam. 0.999	DEL 0.00	32.0	0.02 (Don.L)	3*,13*
c.2215C>T	p.Gln739Ter	Not Present	Not Present	N/A	N/A	39.0	0.00	6,7,14

Variant nomenclature based on transcript ENST00000359655.4.

* Signifies a compound heterozygous variant.

Abbreviations. **PolyPhen2:** Prob.Dam, Probably Damaging; Poss. Dam., Possibly Damaging. **SIFT:** DEL, Deleterious; TOL, Tolerated. **SpliceAI**; Acc.L, Acceptor Loss; Don.G, Donor Gain; Don.L, Donor Loss.

## Data Availability

The datasets generated and analysed during the current study are available from the corresponding authors on reasonable request.
